# Evaluation of QuitNow Men: An Online, Men-Centered Smoking Cessation Intervention

**DOI:** 10.2196/jmir.5076

**Published:** 2016-04-20

**Authors:** Joan L Bottorff, John L Oliffe, Gayl Sarbit, Paul Sharp, Cristina M Caperchione, Leanne M Currie, Jonathan Schmid, Martha H Mackay, Sean Stolp

**Affiliations:** ^1^ Institute for Healthy Living and Chronic Disease Prevention University of British Columbia Kelowna, BC Canada; ^2^ Australian Catholic University Faculty of Health Sciences Melbourne Australia; ^3^ School of Nursing Faculty of Applied Science University of British Columbia Vancouver, BC Canada; ^4^ School of Health and Exercise Science Faculty of Health and Social Development University of British Columbia Kelowna, BC Canada; ^5^ Context Ltd Vancouver, BC Canada

**Keywords:** smoking cessation, web-based health promotion, internet, masculinity, men's health

## Abstract

**Background:**

Men continue to smoke cigarettes in greater numbers than women. There is growing evidence for the value of developing targeted, men-centered health promotion programs. However, few smoking cessation interventions have been designed for men. A gender-specific website, QuitNow Men, was developed based on focus group interview findings, stakeholder feedback, and evidence-based cessation strategies. The website was designed to incorporate a masculine look and feel through the use of images, direct language, and interactive content. Usability experts and end-users provided feedback on navigation and functionality of the website prior to pilot testing.

**Objectives:**

The objectives of the pilot study were to describe (1) men’s use and evaluations of the interactive resources and information on the QuitNow Men website, and (2) the potential of QuitNow Men to engage men in reducing and quitting smoking.

**Methods:**

A one-group, pretest-posttest study design was used. Men who were interested in quitting were recruited and invited to use the website over a 6-month period. Data were collected via online questionnaires at baseline, 3-month, and 6-month follow-up. A total of 117 men completed the baseline survey. Over half of those (67/117, 57.3%) completed both follow-up surveys.

**Results:**

At baseline, participants (N=117) had been smoking for an average of 24 years (SD 12.1) and smoked on average 15 cigarettes a day (SD 7.4). The majority had not previously used a quit smoking website (103/117, 88.0%) or websites focused on men’s health (105/117, 89.7%). At the 6-month follow-up, the majority of men used the QuitNow Men website at least once (64/67, 96%). Among the 64 users, 29 (43%) reported using the website more than 6 times. The men using QuitNow Men agreed or strongly agreed that the website was easy to use (51/64, 80%), the design and images were appealing (42/64, 66%), they intended to continue to use the website (42/64, 66%), and that they would recommend QuitNow Men to others who wanted to quit (46/64, 72%). Participants reported using an average of 8.76 (SD 4.08) of the 15 resources available on the website. At 6-month follow-up, 16 of the 67 participants (24%) had quit, 27 (40%) had reduced their smoking and 24 (36%) had not changed their smoking habits. Repeated measures general linear model showed a significant decrease in the number of cigarettes smoked between the 3-month and 6-month follow-up ( *F*_1,63_=6.41, *P*=.01, eta squared=0.09). Number of resources used on the website, quit confidence, nicotine dependence and age significantly predicted number of quit attempts by those still smoking at 6 months ( *F*_4,45_=2.73, *P*=.04), with number of resources used being the strongest predictor ( *P*=.02).

**Conclusions:**

The results of this research support efforts to integrate gender-sensitive approaches in smoking cessation interventions and indicate that this novel Web-based resource has potential in supporting men’s smoking cessation efforts.

## Introduction

Tobacco use remains a serious and persistent health risk and is a leading preventable cause of death related to cancer, heart disease, and other chronic diseases [ 1, 2]. Since most tobacco is consumed in the form of manufactured cigarettes, this risk is highest among men because they smoke in greater numbers [ 3]. By estimates that include 21 countries, there are over 879 million current smokers, of whom 721 million are men [ 4].

Despite decreases in smoking rates in Canada over the past decade, men (18%) continue to smoke in greater numbers than women (14%) [ 5]. These trends point to a critical need to rethink how cessation services are delivered to men. Although there is increasing evidence for the effectiveness of gender-specific health promotion programs, few men-centered tobacco reduction and cessation interventions have been developed and/or formally evaluated [ 6, 7].

Experts suggest that dynamic and interactive Web-based technologies have the potential to encourage and assist men in accessing and using health promotion information [ 8, 9]. Although findings from a systematic review of Web-based smoking cessation programs indicate their potential usefulness [ 10], the majority of programs have not targeted specific subgroups of individuals or tailored interventions to their preferences and personal characteristics. Evidence suggests that online “one-size fits-all” cessation programs do not garner better outcomes than usual care, printed self-help materials, or email notification interventions [ 11, 12].

Current research reveals men turn to the Internet for health information [ 8, 9, 13]. Websites and social networks influence men’s lifestyles, enable information-gathering, and provide opportunities to engage in mutual help and content co-creation [ 14, 15]. Specific to smoking cessation, Web-based and mobile apps have emerged as commonplace, making possible on-demand and just-in-time information retrieval [ 16- 18]. This, in turn, fosters “collective intelligence,” drawing on men’s preferences for autonomy in decision making related to mapping and monitoring their quit strategies. There is also evidence signaling the effectiveness of tailored, interactive online cessation programs. A three-arm randomized trial (N=1236) conducted in the United States compared the psychosocial variables mediating 30-day abstinence among adult participants assigned to either a basic cessation website, an interactive cessation website enhanced with online social networking, or an interactive and enhanced website with the addition of telephone counseling [ 19]. Increased levels of website utilization predicted higher abstinence rates. However, the authors caution that more work is needed to enhance the effectiveness of online smoking cessation resources [ 19].

To assist with reducing the number of men who smoke, we designed a men-centered smoking cessation website in collaboration with experts from QuitNow, the British Columbia Lung Association’s QuitNow.ca site. The purpose of the current paper is to (1) describe the development and usability testing of QuitNow Men, a novel, tailored, evidence-based smoking cessation website optimized for use on desktop and mobile devices, and (2) report on a pilot study conducted to garner insights about the acceptability and potential effectiveness of the QuitNow Men website. The pilot study objectives were (1) to describe men’s use and evaluation of the interactive resources and information included on the QuitNow Men website, and (2) to evaluate the potential of the QuitNow Men website to engage men in reducing and quitting smoking. The study protocol for all phases of this project was approved by the University of British Columbia Behavioural Research Ethics Board.

## Methods

### Initial Development of the QuitNow Men Website

Four focus groups were conducted with men who smoke or had recently quit (N=56) to gather information on men’s motivations for quitting and their suggestions for developing men-centered resources [ 20]. The men were recruited from three locations in British Columbia, Canada, using posters and online strategies, and represented diverse sociodemographic and ethnocultural backgrounds. Their suggestions for the kinds of support they would find helpful to quit smoking, along with evidence-based cessation strategies, guided the development of gender-specific online resources for the website. QuitNow Men was optimized for mobile use and designed to incorporate a look and feel that would appeal to men 18-45 years of age by using masculine images, direct language, and content that included interactive video dramas [ 21] (see Table 1). In addition, to tailor content to men’s preferences, we used strong, positive messages to promote change (eg, “Put these tactics and tools to good use to get the job done”); connected positive identities, such as being healthy and strong, with being smoke free; and included men’s stories about quitting to show common challenges and create a community of mutual help. An interactive discussion forum, a section for sharing stories about reducing and quitting, and a formatted email to “get a buddy on board” were developed for men to engage each other, provide support, and share personal experiences. To reflect preferences for autonomous decision making, the website was structured to offer men choices by providing an array of resources to map, monitor, and maintain their quit. A section with tactics and tools to support cessation efforts was provided along with quizzes, videos, expert chat, text support, and a smoking calculator. Table 1provides an overview of resources available on the QuitNow Men website.

**Table 1:** QuitNow Men website resources.

Resource		Description
**Man-friendly informational resources**		
	Tactics for getting started	Man-tailored information about different quitting methods, strategies for dealing with withdrawal, cravings, and smoking triggers.
	Tactics for surviving quit day	Man-tailored information about lining up support, gathering supplies, avoiding risky situations, staying busy, and changing routines.
	Tactics for staying on track	Man-tailored information with suggestions for staying smoke free. Topics include slips vs relapses, keeping one’s guard up, and strategies for dealing with stress.
	Patches, gum, and more	Information about quit aids and where to obtain them, including; nicotine patches, gum, inhalers, lozenges, mouth sprays, and prescription drugs.
	Videos on addiction	Inspiring, informational, and motivating videos from the Web on the science behind addiction.
**Man-friendly interactive resources**		
	Pick a quit date tool	Participants select a quit date that will appear in the top corner of the webpage and a digital timer begins a countdown to the chosen date. Emails are sent to the participants: two before the quit date, one on the quit date, and four follow-up emails after the quit date. Once a quit date is reached, the timer begins to count the number of day, hours, and minutes of being smoke free.
	Smoking dependence mini-quiz	A 6-item self-report questionnaire that evaluates participant’s addiction level [ 19] and provides suggestions for next steps.
	Smoking cost calculator	A smoking calculator that calculates the cost spent on cigarettes and provides suggestions of things that money could be used to buy (eg, a new truck). Cost savings are also calculated based on a 50% reduction in smoking behavior.
	If I were Nick (interactive video drama)	Seven brief scenarios about a character, Nick, on his first day of a quit. The audience is encouraged to put themselves in Nick’s shoes and answer reflective questions on how they would think and feel in certain situations [ 18].
**Social support**		
	Tune into the forum	A community forum where participants can connect and discuss topics surrounding reducing and quitting smoking.
	Read shared stories	A space where men can read and share quit stories with other users on the website.
	Text messages with quit tips	A 3-month automated text messaging system that is designed to provide advice and tips to help participants prepare for a quit and stay smoke free. Information is tailored to the specific quit date provided.
	Challenge a friend to quit	Participants are encouraged to challenge a friend to quit with them. Participants are provided with a sample email that can be sent to a buddy through the Web app.
	Expert chat (online quit coach)	Trained quit coaches, available 7 days a week to answer questions or provide advice through a live chat room.
	Quitline (talk to a quit coach)	A toll-free one-on-one consultation with a trained quit coach designed to help participants prepare for a quit and stay smoke free. Following the initial contact, the quit coach schedules five follow-up calls.

### Usability Testing

Prior to launching the pilot study, usability testing was conducted by usability experts (n=4) and with end-users (n=9) to obtain feedback on the navigation and functionality of the QuitNow Men website. We focused on uncovering usability issues related to well-known design principles, as well as those that could impact the end-user’s overall ability to navigate the website, understand the feedback received, and progress successfully through a range of resources. Four usability experts conducted an expert review in pairs and then conducted a “cognitive walkthrough” using two target population personas and scenarios: (1) a man working in an outdoor job who would be using a smartphone, and (2) a man working in a desk job who would be using a desktop computer. A cognitive walkthrough is a specific method of expert review, in which the reviewer completes a series of tasks that the interface is meant to support from the perspective of a target user [ 22]. Potential issues related to expected end-user actions and associated cues/feedback were identified and categorized as being issues related to either navigation, information architecture, or functionality. Issues were rated on a scale of 1-4 ranging from suggestions/opportunities for improvement to urgent usability problems. A total of 27 issues were identified, which developers addressed through revisions to the website resource.

End-user usability testing was then conducted with 9 men using iOS and Android smartphones, a laptop, and a desktop computer. The men were recruited using e-postings on social media. A think-aloud protocol was followed, using Morae to capture audio and video of device screens as men completed tasks related to the expected use of the system (eg, enter quit date, calculate how much smoking has cost you so far). Recordings were analyzed and violations of usability principles were again classified based on usability best practices [ 23]. The main issues were related to not recognizing that the QuitNow Men logo was the method to return to the home page (recognition not recall), not having a back button on some pages (user control and freedom), and mixed use of first and third person in the text (match between system and real world). After the tasks were completed, the majority of the men (8/9, 89%) also completed a short questionnaire evaluating the usability of the website. Every man agreed or strongly agreed that the website was easy to use, and the majority (7/8, 88%) felt very confident using the website. Half of the men agreed or strongly agreed with the statement, “I think that I would like to use this website frequently.” Participants also indicated a preference for content on the site to be phrased in the first person (eg, How much cash am I blowing on smoking? How dependent am I?), and the website was modified accordingly. The QuitNow Men website was then readied for pilot testing.

### Pilot Test

#### Study Design and Sample

A one-group, pretest-posttest study design was used. Men were recruited using social media (Facebook and Twitter) and online classified advertisements (Kijiji, Craigslist, and Castanet) between June 26, 2014, and September 8, 2014. Eligible participants were male, lived in Canada, read and understood English, had a valid email address and Internet access, and currently smoked but were interested in quitting. Participants provided online informed consent prior to participation.

#### Study Procedures

All baseline and follow-up data were collected using online questionnaires administered through a secure, password-protected link. Upon completion of the baseline questionnaire, participants were provided with a unique ID and password to access the QuitNow Men website and asked to interact with the site over a 6-month period. Study participants were invited to complete follow-up questionnaires at 3 months and 6 months. Two subsequent emails were sent to non-responders at 1-week intervals reminding them to complete the questionnaire. Two intervention emails were sent out between the 3-month and 6-month follow-ups to remind participants to continue to use the website. Intervention emails included a humorous anecdote to encourage participants to return to the site as well as a link to the homepage. Participants received a maximum of CAN $50 for participating in the study ($25 gift card for completing the 3-month follow-up and $25 gift card for completing the 6-month follow-up).

### Measures

#### Baseline Measures

Participants were asked about their current use of technology and preferences for connecting to the Internet, as well as previous use and interest in using online smoking cessation resources. Questions were included to gather data on smoking patterns, and dependence was measured using the Fagerstom Test for Nicotine Dependence [ 24], a standardized instrument for assessing intensity of physical addition to nicotine. Self-efficacy to avoid smoking temptation across various situations (eg, at a party, when under stress) was assessed using Velicer et al’s [ 25] instrument in its 9-item version [ 26]. Cronbach alpha for this measure was acceptable for this sample (alpha=.810). Demographic data were also collected including age, education level, and marital status.

#### Follow-Up Measures

##### Usability and Acceptability

Participants reported on the number of visits to the website between baseline and 3 months, and between 3-month and 6-month follow-up, with the following options: not at all, once, 2-3 times, 4-6 times, more than 6 times. For each resource they reported using, men were asked to rate its helpfulness using a 3-point scale (not helpful, somewhat helpful, very helpful). Acceptability of the website was measured by assessing level of agreement with 4 statements using a 5-point Likert response format (strongly disagree to strongly agree): “QuitNow Men is easy to use,” “The design and images used on QuitNow Men are appealing,” “I intend to continue to use QuitNow Men resource,” and “I will recommend QuitNow Men to other men who want to quit smoking.”

##### Smoking Behavior

Participants were asked if they currently smoked daily, occasionally, or not at all. Those who smoked daily or occasionally were asked about number of quit attempts lasting at least 24 hours in the past 3 months, number of cigarettes per day on the days that they smoked, readiness to quit, and confidence in ability to quit during the next month. Readiness to quit was measured using the Contemplation Ladder [ 27], which assesses a smoker’s position on a continuum ranging from no thought of quitting (0) to being engaged in taking action to quit (10). Finally, confidence in smoking cessation was measured using a single item that asked: “If you decided to quit smoking during the next month, how confident are you that you could do it?” Responses to this item were coded “not at all confident”=1, “not very confident”=2, “somewhat confident”=3, and “very confident”= 4.

### Data Analysis

All data were analyzed using SPSS version 22. Descriptive statistics were used to describe website usage and participant evaluations of QuitNow Men. One-way analysis of variance (ANOVA) was conducted to determine if those who dropped out of the study significantly differed in their demographic profile from those who completed both 3-month and 6-month follow-up. Repeated-measures general linear model, assuming compound symmetry, was used to investigate within-subject differences between 3-month and 6-month follow-ups on number of cigarettes smoked, quit confidence, and readiness to quit score. One-way ANOVA was conducted to assess whether or not those who quit smoking, reduced smoking, or had no change in smoking status at 6 months differed on number of website resources used at 6-month follow up. Finally, multiple logistic regression was use to investigate predictors of the number of quit attempts at 6 months. Standardized predictor variables included number of resources used on the website (at 6-month follow-up), number of visits to the website, quit confidence (at 6-month follow-up), nicotine dependence, numbers of years smoking, and age.

## Results

### Sample

A total of 117 men completed the baseline survey of which 67 (57.3%) completed both the 3-month and 6-month follow-up survey. The 117 men had an average age of 39.82 (SD 11.08) with a range of 21-68 years. The majority of the men (78/117, 66.7%) were 45 years of age or younger. Sample demographic characteristics at baseline are provided in Table 2. At baseline, participants reported smoking for an average of 24 years (range 2-55, SD 12.12) and smoked on average 15 cigarettes per day (range 3-40, SD 7.4). Based on Fagerstrom scores, 48% were classified as minimally dependent, followed by 27% moderately dependent, and 25% highly dependent. The average smoking self-efficacy/temptation score was 2.7 out of a possible 5, where higher scores demonstrate lower levels of self-efficacy (higher temptation). Participants reported spending an average of 2.8 hours per day on the Internet, the majority connected to the Internet via home computer (105/117, 89.7%), followed by a smartphone (89/117, 76.1%). The majority of participants (103/117, 88.0%) had not previously used a quit smoking website or websites that focus on men’s health (105/117, 89.7%). A statistically significant age difference was found between those who completed the follow-up surveys and those who dropped out ( *F*_1,113_=6.75, *P=*.01, eta squared=0.056). Those who dropped out were significantly older (mean 43.12, SD 11.53, range 23-68) than those who completed follow-up measures (mean 37.83, SD 10.18, range 21-58). As well, those who dropped out had been smoking for significantly longer than those who completed follow-up ( *F*_1,113_=8.16, *P=*.005, eta squared=0.067). Yet there were no significant differences on Fagerstrom scores ( *P*=.84), average number of hours per day on the Internet ( *P*=.28), number of quit attempts in the past 2 years ( *P*=.67), and self-efficacy/temptation score ( *P=*.70).

**Table 2:** Baseline demographics characteristics (N=117).

Characteristic		n	%
**Age**			
	20-45 years	78	66.7
	>45 years	39	33.3
**Cultural identity**			
	Canadian/Caucasian	85	73
	First Nations/Metis/Inuit	5	4
	Other	17	14
	Missing/Invalid	10	9
**Highest level of education**			
	Incomplete high school	14	12
	Complete high school	42	36
	Complete non-university (vocational, technical, trade)	23	20
	Complete university degree / diploma / certificate	34	29
	Other	4	3
**Marital status**			
	Married	40	35
	Single	39	33
	Common law/live-in partner	24	20
	Divorced or separated	14	12
**Main activity**			
	Working for pay or profit	71	61
	Caring for family and working for pay or profit	19	16
	Recovering from illness or disability	12	10
	Looking for work	6	5
	Going to school	5	4
	Unemployed and not looking for work	2	2
	Caring for family	1	1
	Retired	1	1

### Website Usage and Evaluation of QuitNow Men

**Figure 1:**
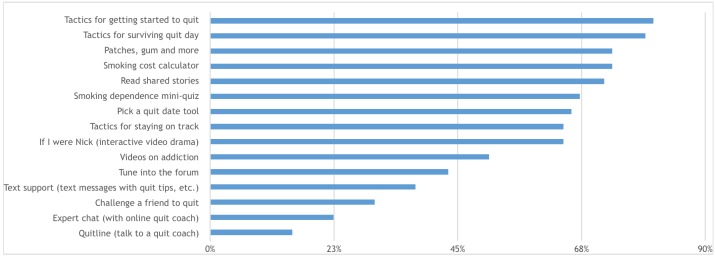
Percentage of participants reporting use of resources on QuitNow Men at 6 months (n=67).

**Figure 2:**
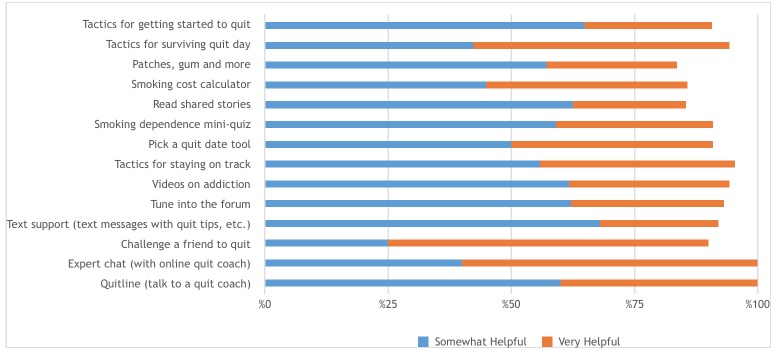
Percentage of participants rating resources they used on QuitNow Men as "somewhat helpful" or "very helpful" (n=64).

Of the 67 men who completed both follow-up surveys, 6-month data showed that 64 men (96%) reported using the QuitNow Men website at least once during the pilot study. Nearly half of the users (29/64, 45%) reported using the website more than 6 times, while 10 (16%) used the website 4-6 times, 24 (38%) used it 2-3 times, and 1 man (2%) used it once. At 6-month follow-up, the majority of users (45/64, 70%) reported that they were either satisfied or very satisfied with the website. In addition, they agreed or strongly agreed that the website was easy to use (51/64, 80%), the design and images were appealing (42/64, 66%), they intended to continue to use the resource (42/64, 66%), and that they would recommend QuitNow Men to others who wanted to quit (46/64, 72%). Participants reported using an average of 8.76 (SD 4.08) of the 15 resources available on the website. Figure 1details the percentage of participants who reported using each of the QuitNow Men resources during the 6 months. Figure 2shows men’s ratings of the helpfulness of the resources that were used.

### Use of QuitNow Men and Smoking Behavior

Self-reported smoking status at 6-month follow-up indicated that 16 of the 67 participants (24%) had quit, 27 (40%) had reduced their smoking, and 24 (36%) had not changed their smoking habits. Among those 51 men who were still smoking at 6-month follow-up, 44 (86%) reported that they had quit for 24 hours or longer since using the QuitNow Men website, with an average number of quit attempts of 3.38 (SD 2.33). The average readiness to quit score among those still smoking at 6-month follow-up was 7.61 out of a possible 10. The majority (39/51, 77%) were somewhat confident or very confident that they could quit smoking during the next month, with no significant differences between 3-month and 6-month follow-up. Repeated measures general linear model showed a significant reduction in the average number of cigarettes smoked between 3-month and 6-month follow-up ( *F*_1,63_=6.41, *P=*.01, eta squared=0.09). At 3-month follow-up, the average number of cigarettes smoked was 6.84 (95% CI 5.24-8.43) and at 6-month was 5.25 (95% CI 3.82-6.68).

Those who quit smoking, reduced smoking, or had no change in smoking status at 6 months differed on the total number of website resources used ( *F*_2,64_=3.34, *P=*.042, eta squared=0.094). Post-hoc least significant difference analysis showed that men who reduced the number of cigarettes smoked used significantly more website resources than those that had no change between time points ( *P=*.024). Means and confidence intervals can be seen in Table 3.

**Table 3:** Number of website resources used by smoking status.

Smoking status	Number of website resources used	
	Mean	95% CI
Quit smoking	7.25	5.52-8.98
Reduced smoking	10.0	8.37-11.63 ^a^
No change in smoking	7.31	5.20-9.43 ^a^

^a^ *P=*.03.

Multiple logistic regression was conducted to identify if number of resources used on the website, number of website visits, quit confidence, nicotine dependence, and participant age predicted the number of quit attempts among men who were smoking at 6-month follow-up. Potential predictors were converted to Z scores. Multicollinearity diagnostics showed that the number of resources used on the website and website visits were highly correlated ( *r*_s51_=65.9, *P*<.001) resulting in unacceptably low tolerance levels (<.865; 1-R ^2^). It was determined that examining the number of resources used on the website would be more representative of examining the number of times the user visited the website and as such, website visits was removed from the regression (see Table 4). The combination of remaining variables significantly predicted number of quit attempts ( *F*_4,45_=2.73, *P*=.04). The adjusted R ^2^value was 0.124. Number of website resources used was the strongest predictor of quit attempts ( *P*=.02).

**Table 4:** Logistic regression predicting quit attempts (6-month follow-up).

Variable	B (CI)	SE	β	*P*
Number of resources used on website at 6-month follow-up	.81 (0.12-1.50)	0.34	.35	.02
Quit confidence at 6-month follow-up	.50 (1.2-0.20)	0.35	.21	.16
Nicotine dependence	.33 (-0.33 to 1.0)	0.33	.15	.32
Age	.58 (-0.14 to 1.31)	0.36	.23	.11
Constant	3.34 (2.71-3.96)	0.31		<.001

## Discussion

### Principal Findings

This work adds to the small but growing field of online health promotion innovations directed specifically to promote men’s health. To our knowledge, QuitNow Men is the first men-centered smoking cessation website in the world, and the pilot study findings reveal that the site is appealing to men who want to quit and demonstrates potential as a self-guided smoking cessation resource. The majority of men reported they had quit smoking for 24 hours or longer since using the QuitNow Men website, and the number of resources used on the website and quit confidence predicted the number of quit attempts. At the 6-month follow-up, 24% reported having quit smoking. Although QuitNow Men was designed for adult men 45 years and younger, men over 45 years of age were interested in using this online resource suggesting that this approach to supporting smoking cessation may have broad appeal among men. Since older men were more likely to drop out of the study, it is possible QuitNow Men did not have the same appeal for this group given that images, text, etc, were designed for a younger audience. Nevertheless, given older men’s interest in trying this online resource, it is worth considering how an online smoking cessation resource could be tailored to reflect the life stage and needs of this group and what additional supports they may require. It should also be noted that among those men that remained in the study, the oldest was 58 years of age and multiple regression results show that age was not a significant predictor of number of quit attempts made. Overall, in the broader context of men’s utilization of eHealth apps, these results afford important empirical insights that could guide future online smoking cessation programs and provide direction to other eHealth resources aimed at reducing men’s health risks (eg, abstaining from high-fat foods, addressing alcohol overuse).

In this study, the 64 men who completed both the 3-month and 6-month follow-up surveys and used the resources on QuitNow Men rated them as helpful. This suggests that the specific design elements used in the QuitNow Men website hold value for increasing and improving men’s engagement in the core components of this self-guided intervention. For example, setting up the website to allow for quick exploration of a number of resources and strategies (as distinct from in-depth exploration) was a design element used to entice men to learn about a variety of approaches. In turn, the finding that exploring more resources increased men’s likelihood of having made a quit attempt suggests that the variety of resources included on QuitNow Men may be a key factor in the overall positive response to the site. It is interesting to note two of the least-used resources (ie, expert chat and the quitline) were the highest rated in terms of helpfulness among men who used them. Although few men desired personal contact with experts either through chat sessions or the quitline, these supplementary delivery modes provide an important component of Internet smoking cessation programs for men given that they offer a degree of anonymity and convenience (in comparison to face-to-face counseling) and have been found to be effective in providing proactive support to smokers [ 28, 29]. In addition, there is empirical support for the value of including multiple modes of delivery in Internet programs to promote health behavior change [ 30, 31]. However, determining the optimal balance between self-management resources and interactive components, as well as the relationship of activity complexity to cessation rates, warrants further investigation with a larger sample.

More broadly, the findings drawn from this pilot study remind us that the Internet provides a suitable medium for men’s health promotion and, more specifically, for delivering smoking cessation interventions to men. Tobacco reduction and cessation research has revealed strong evidence supporting therapist-led interventions, and intensive group and individual counseling interventions to assist with cessation [ 32- 34]. These intensive interventions, however, are often dependent on primary healthcare professionals delivering or facilitating the sessions at a specific time and place, which is often inconvenient to individuals. As a result, the reach of this type of intervention is limited [ 35]. Although Internet-based smoking cessation interventions offer a low cost, accessible option, and have been found to be acceptable to users and effective in aiding cessation [ 36], we have found through experiences in our region that the majority of users of an online cessation resource for the general population are women. The QuitNow Men smoking cessation website is a promising and potentially powerful resource toward balancing this gender inequity and engaging men in taking actions to become smoke free. Determining the best means to promote the use of this novel program to male smokers will be important.

### Limitations

The findings of this study should be considered in light of several limitations. The sample of men that participated in this study may not be representative of all male smokers. In addition, self-report measures (eg, with respect to smoking patterns, quit attempts) may have introduced recall and reporting bias. Smoking cessation outcomes were not biochemically verified. Nevertheless, the findings provide important estimates of outcomes that reflect the potential value of the QuitNow Men smoking cessation website and a basis for conducting a full-scale evaluation.

### Conclusion

The results of this research support efforts to integrate gender-sensitive approaches in health promotion interventions. The QuitNow Men smoking cessation website is highly acceptable and engaging to men interested in reducing and quitting smoking. Results indicate that this novel resource, tailored to men, has potential to support and perhaps catalyze men’s smoking cessation efforts. Furthermore, given the tailored nature of the QuitNow Men smoking cessation website and that it caters to the specific values of a particular population (ie, men) [ 37- 39], it has the potential to attract, engage, and retain men interested in quitting smoking.
